# The Sarrazin effect: the presence of absurd statements in conspiracy theories makes canonical information less plausible

**DOI:** 10.3389/fpsyg.2013.00453

**Published:** 2013-07-18

**Authors:** Marius Hans Raab, Nikolas Auer, Stefan A. Ortlieb, Claus-Christian Carbon

**Affiliations:** Department of General Psychology and Methodology, University of BambergBamberg, Germany

**Keywords:** conspiracy theories, narrative construction, adaptation, liking, preference, external validity, reframing, assimilation

## Abstract

Reptile prime ministers and flying Nazi saucers—extreme and sometimes off-wall conclusion are typical ingredients of conspiracy theories. While individual differences are a common research topic concerning conspiracy theories, the role of extreme statements in the process of acquiring and passing on conspiratorial stories has not been regarded in an experimental design so far. We identified six morphological components of conspiracy theories empirically. On the basis of these content categories a set of narrative elements for a 9/11 story was compiled. These elements varied systematically in terms of conspiratorial allegation, i.e., they contained official statements concerning the events of 9/11, statements alleging to a conspiracy limited in time and space as well as extreme statements indicating an all-encompassing cover-up. Using the method of narrative construction, 30 people were given a set of cards with these statements and asked to construct the course of events of 9/11 they deem most plausible. When extreme statements were present in the set, the resulting stories were more conspiratorial; the number of official statements included in the narrative dropped significantly, whereas the self-assessment of the story's plausibility did not differ between conditions. This indicates that blatant statements in a pool of information foster the synthesis of conspiracy theories on an individual level. By relating these findings to one of Germany's most successful (and controversial) non-fiction books, we refer to the real-world dangers of this effect.

## Introduction

A government arms the nation's most prominent skyscrapers with explosives and directs passenger planes right into these buildings: taken at face value, such an evil scenario seems completely off-wall. However, such accusations are a common, probably a typical ingredient of conspiracy theories. While a government trying to conceal acts of failure—for example, the underestimation of a terrorist threat—might be seen in the realm of possibility, the widespread acceptance of very complex malicious plots, such as a government deliberately killing thousands of the own people, is a challenge for psychology. On the one hand, we need to understand why many people adhere to a world view which implies permanent threat to every individual (including themselves). On the other hand, disturbing revelations—such as the recent *PRISM*^1^ leak—make it clear that denying global conspiracies *per se* would be ignorant.

This challenge has multiple theoretical as well as methodological aspects: how and why does the presence of quite extreme information influences the processes of opinion formation? How can this process be captured and investigated in a valid and yet standardized way? And how can research that addresses these processes take a non-arbitrary stance in the assessment of an individual's conspiracy beliefs, when there is no clear distinction between true and false?

There have been various research efforts on individual differences in the endorsement of conspiracy theories (e.g., Swami et al., 2010). There is a finding that people are willing to adopt obviously contradictory conspiratorial facts at the same time (Wood et al., 2012). Lewandowsky et al. (2013b) indicate that belief in one conspiracy theory is correlated with the belief of other theories. Swami and Coles (2010) provide a comprehensive overview of research on this subject. The pro-active and constructive aspect of *creating* a (conspiracy) theory, however, has not been regarded in an experimental design so far.

The analysis of documents like websites and books is appealing, but still has also clear limitations, as we cannot take for granted that these published theories are representative for the stories the majority of people would adopt. Millions of people around the globe create, compile, process, discuss, and reproduce conspiracy theories not only on internet platforms, private websites or blogs, but also in personal communication, which is hard to assess in research. We assume these people to be active information seekers who construct views on important events that match their beliefs and values; and whose beliefs are in turn influenced by information. Extreme theories, in books as well as on the web, would serve as a *mixed bag*, that (speaking with P.T. Barnum) offer “something for everyone”; so everybody is free to adopt some story fragments only. As we have no further information about and control of the regarding creators, proliferators and consumers of such content we need methods—in addition to content analysis (e.g., Lewandowsky et al., 2013a), interviews (e.g., Sapountzis and Condor, 2013) and standardized questionnaires (e.g., Lewandowsky et al., 2013b)—which allow for the dynamic character of compiling, reframing and linking of information to unfold.

Here, we suggest the method of *narrative construction* as a new means to explore the multi-facet phenomenon of conspiracy theories. It allows an individual to construct a *story* for a given event (e.g., the terrorist attacks of 9/11) by selecting and compiling pieces of information related to this event from different content categories. By doing so, we can assess how much conspiracy an individual assumes to be at work concerning the event; without compelling the researcher to define what a “true” story looks like.

This article consists of two main parts. In the first part, we present an exploratory study that helped us to identify core constituents of conspiracy theories in a bottom-up approach. Subsequently, these constituents were used as templates, for pieces of information about 9/11 (retrieved from the World Wide Web). We compiled two sets of information: one set with official and mildly conspiratorial (i.e., with limitations in space and time) information and another set that comprised additional extremely conspiratorial statements. In a laboratory setting test subjects were asked to construct a plausible story of the events of 9/11 using one of these sets of information. This main study showed that the presence of extreme information induced a significant shift of the resulting stories toward a conspiracy theory; importantly, this shift was not paid for by lower plausibility as shown by ratings each test subject gave afterwards for his/her story.

In the second part of this article, we discuss a recent public debate on Sarrazin's (2010) book *Deutschland schafft sich ab* (*Germany is abolishing itself*) in the light of these findings. The book is among the most successful non-fiction works of the past decade in Germany, (in-) famous for its polemic portrayal of Islamic culture (Sarrazin had been prominent before this debate as senator of finances in Berlin from 2002 to 2009 and as member of the Executive Board of the Deutsche Bundesbank until 2010). Sarrazin's book was our point of origin: Not only was its impact on political discourse huge; the author presented a patchwork mixture of established facts, assumptions, wild speculations and polemic accusations. We consider his book, at least compatible with conspiracy theories, if not even a conspiracy theory on its own, as we will discuss later on.

If the presence of extreme statements in a pool of given information seduces people to disregard standard information, conspiracy theories can be dangerous indeed: It may shift the tenor of public debate and the individual's judgments of plausibility toward the extreme.

## Materials and methods

### Rationale for using the method of narrative construction

In spite of the numerous attempts to define what a conspiracy theory is [e.g., Grüter, 2010, even dedicates a full monograph to this question], we found it hard to derive distinct categories of elements from any of such definitions. Many authors refer to the definition of Hofstadter (1965), who claims that a conspiracy theory of a vast, sinister and yet subtle machinery of influence to destroy a way of life. This sums up the main features of common conspiracies, but is too vague to allow for the generation of distinct narrative elements (Bale, 2007). Bale confines himself to political conspiracies aiming at a more differentiated definition. However, he presents discriminative features that mainly define conspiracies by the attributes of the conspiring force. In other words, he discusses a conspiracy's characteristics, which is not the same as a conspiracy theory's narrative parts. Additionally, we think that such an attempt would run the danger that primarily the well-known and mostly extreme conspiracy theories—the ones that were used to generate the definitions—become paradigmatic. Research would then focus on such extremes while missing the subtle shadings and nuances of individual theories and everyday phenomena.

In his analysis of Russian folk tales, Propp (1972) has already pointed to the problems of a classification without a guiding principle for defining a story's features. His solution was a bottom-up categorization of 100 folk stories. He discriminated the tales' contents and the narrative functions of the elements he found and finally arrived at seven essential story elements (like, the *Hero* or the *Adversary*). Thus, to identify the morphological constants of conspiracy theories, we decided to take a bottom-up empirical stance.

### Prerequisite: a bottom-up assembly of conspiracy theory building blocks

In a preliminary study, we determined which elements are likely to constitute a conspiracy theory. Major aim was to collect maximally diverse kinds of such theories. Five interviewers asked 38 people (students from the University of Bamberg, their friends and relatives) which “intrigues and secret schemes, for example conspiracy theories” they know of. Afterwards, we asked them to reproduce their “favorite conspiracy theory” by their own words. The interviewers also wanted to know where they had heard this story, and why it is their favorite conspiracy theory. As a next step, we asked “which elements are part of most conspiracy theories” as open question, recording the answers verbatim.

The recorded material formed the basis for a bottom-up process of categorization. Each interviewer tried to rephrase the answers from another interviewer's participants on a more abstract level. The derived categories had then to be defended in an argument with the other interviewers. This kind of argumentative validation, as described by Mayring (2005), went on until all interviewers agreed on a set of six categories for “elements of conspiracy theories,” including category definitions. Due to this inductive process, not all categories are strictly homogenous; however, a further subdivision of categories could not be justified in the argumentative process based on the given data.

In order to evaluate the importance of these basic items of conspiracy theories, we printed them on cards (one category along with definition and examples on each card) and handed out shuffled sets (each set containing all elements) to 28 participants (undergraduate students of psychology, 23 female, *M*_age_ = 19.7 years) which had not participated in the initial interviews. The participants were asked to rank these elements by “laying out the cards in the order of subjective importance” and to write down the rank of each item on the respective card when finished. We aggregated these ratings by ordering the items according to the mode of rank orders. The bottom-up generated categories were *odd event*, *evidence*, *non-transparency*, *publicity*, *group of conspirers,* and *myth* (enlisted in Table 1).

**Table 1:** **Items generated by a bottom-up process of categorization, ordered descending by importance**.

**Category label**	**Category definition**	**Standard examples**
Odd event	There is a relevant event that gains interest of many people. There are some open questions concerning this event	• “Apollo-mission”
		• “9/11”
		• “Kennedy assassination”
Evidence	There is evidence, observations, artefacts, and other indications, that are used by conspiracy theorists to support their theories. There are secret signs and symbols supporting the conspiracy theorists' view	• “Symbols seen everywhere”
		• “Undeniable facts”
Non-transparency	The situation about available information concerning a topic is non-transparent. Media coverage is obscure. There is cover-up and manipulation of information	• “Cover-up of reality”
		• “Not enough inside-information available”
Publicity	There is an official viewpoint for a topic. Public agents (e.g., government, experts, scientists, intelligence agencies) acknowledge this viewpoint. However, this account is regarded by some with scepticism and distrust. The official viewpoint contradicts the non-official viewpoint by conspiracy theorists	• “The media spread information”
		• “Experts that testify”
Group of conspirers	There is a group of conspirers. These conspirers are evil and influential, and strive to gain more and more money and power. They forge a secret plot at the expense of other groups or individuals	• “Persons that work in secrecy”
		• “A chosen or intricate minority”
Myth	Historic myths exert a strong influence on conspiracy theories. There are esoteric elements as part of conspiracy theories	• “Esotericism”
		• “A fight between good and evil”

### Method of narrative construction

Our aim was to allow for the idiosyncratic process of constructing a story under controlled and comparable conditions. We developed the method of *narrative construction* that enables us to observe the process, and to quantify each participant's output with regard to the hypothesis.

**Figure 1:**
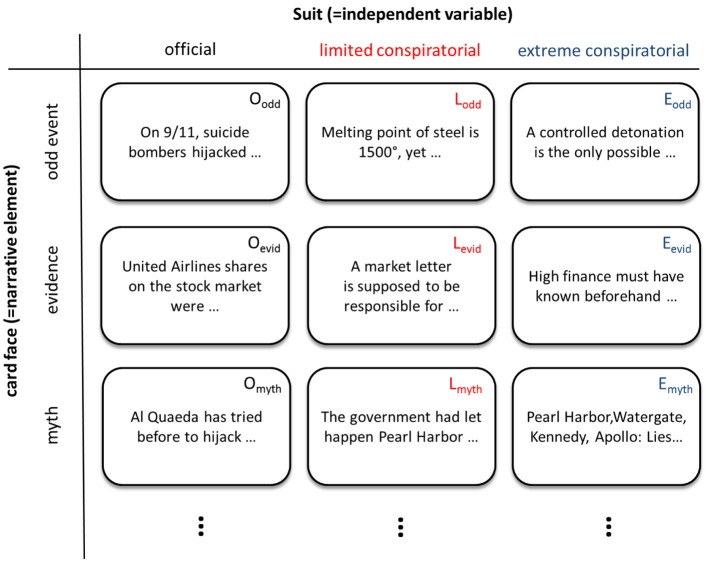
**Overview of the narrative construction design**.

The material for the *narrative construction paradigm* is a compilation of laminated paper cards (each about the size of a playing card, i.e., 10 × 6 cm). These cards are compiled according to the hypotheses in the following manner (exemplified in Figure 1):

- For each independent variable there is a corresponding *suit*, comparable to spades, hearts, diamonds and clubs in a deck of playing cards. For example, if one would be interested to compare internal vs. external attribution in a personal narrative, there would be one suit of cards with statements compatible with internal control beliefs, and one suit with cards all assuming external control. For our case, we compiled one suit containing *official*, one containing *limited conspiratorial*, and one containing *unlimited conspiratorial* items (representing a three-stepped approach).
- Within the suits, there are cards for the categories, i.e., the elements deemed important for the narrative. Each suit contains corresponding cards, like there's an ace of spades, an ace of hearts, etc. For exploring a narrative of control beliefs, there might be one card for work (in the card came metaphor, a king), one for family (say, a queen), one for sports (a joker), etc. (in contrast to playing cards, there is no rank order obvious to the participant). In our case, with six conspiracy theory elements/categories, there should be at least six cards within each suit—one per category, corresponding between suits.
- To allow for more complex narratives, it is possible to compile more than one card per category. For example, one might include three items concerning private life. This is not a feature of playing cards, but can be thought of, e.g., several Queens, all slightly different in their appearance. In conspiracy research, for example more than one card concerning the odd event might be useful

Each participant is handed out the set of shuffled cards at once. They are asked to “construct a story that is—in his personal view—a plausible explanation” for cause for a certain event or process (for example, work-life balance; or, as in our case, 9/11). In the beginning, the participant is instructed to read each card and to coarsely categorize the items into two groups, a “plausible” one and a “not plausible” one. Each of these categorizations could be revised in the course of the process. After the initial pre-sorting, the participant is asked “to serialize the cards to produce a stringent and plausible course of events using as few or as many cards” as he wants. Again, removing or adding cards to the “not plausible”-heap is still, and explicitly, allowed. Furthermore, no time restriction is applied.

By assigning participants to groups and varying the cards between these groups, different research questions can be addressed. To test the influence of the presence of a specific independent variable (= suit), the presence of this suit can be varied. To fathom whether the presence of a specific category influences the selection behavior concerning the other categories, only one group of participants receives cards of this category (for example, Queens present vs. no Queens present).

After the participant has indicated that he/ she is satisfied with his/ her story, he/ she is further asked to rate “how plausible the laid-out story is” with regard to the event in question, on a five-point Likert scale (1 = not plausible, 5 = plausible). Finally, the generated narrative is recorded (by writing down each card's code, printed on the backside that indicates category and factor level in the laid-out order). This procedure is conceptually similar to Meichenbaum's (1996) constructive-narrative therapy which emphasizes the importance to re-construct one's life story when suffering from post-traumatic stress disorder. Wilson (2002) regards introspection as a personal narrative “whereby people construct stories about their lives, much as a biographer would” (p.162). This kind of introspection is seen as beneficial for one's mental well-being by Wilson. He also notes that the process is vulnerable to omissions and simplifications—which are, in our context, not interfering variables, but in fact the effect of interest. McAdams (1997) even argues that we *are* in fact the stories we create.

We devised the narrative construction to be a third way, besides questionnaires and interviews. Already with three dozen cards (for example, three suits à 12 cards), there are billions of possible stories, i.e., combinations. Compared to a questionnaire, this allows for more diversified, idiosyncratic results. Reading, evaluating, sorting and laying out multiple cards can be considered to be more demanding cognitively than serially answering a number of questionnaire items, and it would allow to assess the process of opinion formation, too; for example, by asking participants to *think aloud* while constructing the story. This comes at a price: psychometric criteria can't be applied straightforward here.

Compared to an interview, narrative construction is tighter. The number of items is limited. A transcription and categorization after the experiment is not necessary, as the cards are coded and the chosen items can easily be written down. However, in contrast to an interview, a spontaneous introduction of new items is not possible. The participant's attention stays focused on the process of story creation in narrative construction, while an interview introduces a social facet. It depends on the research question if introducing social interaction is instrumental or a confounder.

A simple evaluation of a narrative construction's result would be to count the number of items chosen from each suit (for example, internal vs. external attribution, when there were two according suits); and/or to count the number of card faces chosen (e.g., how many participants have included “sports” in their work-life-balance narration). This evaluation would be straightforward and could tell which attributional style is predominant in the sample, and/or which aspects are most relevant for people when it comes to balancing their life. By varying specific aspects, the influence some information exerts onto other information can be assessed. For instance, by giving some participants an additional *suit*, the impact of the availability of this information can be measured. Another way is handing out some additional *cards*. Sticking to the control belief example, we could assess how stories change when people are offered cards allowing for counseling or therapeutic advice.

More sophisticated assessments could aim at the structure of stories, e.g., look for typical sequences. Also, one could test if certain aspects nearly always appear together, or turn out to be mutually exclusive.

In sum, whenever a questionnaire seems too rigid, when a thorough and attentive process is desirable, and when narrative *structures* might be relevant, narrative construction might be an option. However, when a dyadic social interaction is preferable, when hypotheses are too vague, and when the topics in focus are too broad to be represented adequately with a deck of cards, an interview should be preferred. Yet, there are research questions where a combination of narrative construction and interview is appealing. By interacting with a deck of topic-related cards, participants might get a grip on a topic, by evaluating all aspects the researcher likes to consider. This sort of elaborate priming might help to facilitate a subsequent interview.

### Constructing a 9/11 story

#### Material

For our research question, we compiled 14 cards for each suit (see Table 1). With respect to the bottom-up derived elements of conspiracy theories: two for *group of conspirers*, one for *non-transparency*, one for *publicity*, three for *odd event*, three for *evidence* and one for *myth*. Each item was present in each suit (i.e., 3 cards), fueled with contents from typical (1) *official*, (2) *limited conspiratorial*, and (3) *unlimited conspiratorial* viewpoints. The *official* suit card always bore a category-related statement that was in accordance with official 9/11 reports and documents (drawing on respectable sources, e.g., governmental reports made public on the internet). For example, an official *group of conspirers*-item was: “9/11 mastermind were Islamist terrorists, led by Osama bin Laden, to attack the detested Western culture.”

The card in the *limited conspiratorial* suit was prepared with an item that contained an explanation describing a conspiracy of moderate strength. Specifically, this level was formed in accordance with Lutter's (2001) categorization of conspiracies, corresponding to a conspiracy limited in time and space. This can also be thought as matching Daniele Ganser's (n.d.) 9/11-view “let it happen on purpose” (LIHOP). In this view, the Bush administration did not initiate the attacks, but knew beforehand and did not take countermeasures. We compiled information from web resources like Wikipedia that matched this level. The “group of conspirers”-item here read: “The US administration had let happen the 9/11 attack to justify the wars in Afghanistan and Iraq.”

In the *unlimited conspiratorial* suit, a card assumed a conspiracy with no clear bounds within space and time, or a “make it happen on purpose” (MIHOP) viewpoint in the sense of Ganser (n.d.). For example, it read: “The US administration had planned and conducted the 9/11 attack to justify the wars in Afghanistan and Iraq.”

So for each of the six categories (odd event, evidence, non-transparency, publicity, group of conspirers and myth), there was at least one triplet of cards (one card with an official statement, one limited conspiratorial and one unlimited conspiratorial); details in Figure 1.

An exempt was a further category, *absurdity,* where all items were completely off-wall: One assumed “thermonuclear devices hidden in the Twin Towers,” one “killer satellites from outer space,” and one stated that “the Syrian newspaper Al Thawra has reported that 4000 Jewish WTC employees were warned beforehand and did not show up on work on 9/11.” These items were identical for both experimental groups, included for another research question and are not considered any further for the hypothesis discussed here.

#### Participants

Thirty persons (26 female, *M*_age_ = 22.4 years, range: 19–55 years) took part in the study. They were recruited at and around the campus of the University of Bamberg; they were naïve to the aim of the study and had not been involved in any other study described in this paper. The participants were randomly assigned to two groups: (1) *modest contents group* and (2) *extreme contents group*.

#### Procedure

The *modest contents group* was handed out a card deck with 29 items, containing the *official* as well as the *limited conspiratorial* suit (plus the three-card subset absurd). The *extreme contents group* received the same deck and additionally the suit with 13 *unlimited conspiratorial* items. All were asked to “construct a plausible story of the events of September 11th 2001, as a single coherent story or consisting of coherent or controversial fragments,” without time restrictions. When the participant had considered the story finished, the chosen items and their layout were written down. The participant was then asked to rate “how plausible the 9/11 story version just laid out is” on a five-point Likert scale (among other questions related to other hypotheses). Overall, the participants spend 21 min on average to construct their story, with a range from 8 to well over 30 min.

## Results

The groups did not differ significantly in terms of age [*M*_modestgroup_ = 21.3, *M*_extremeroup_ = 24.8, *F*_(1, 28)_ = 2.17, *p* = 0.15, *n.s.*]. Each group consisted of 13 female and two male participants.

To compare the stories between groups, we summed up the number of cards chosen from each conspiratorial level (official, limited and unlimited) over all categories. So for each participant, we added up all official items, all limited conspiratorial items and all unlimited conspiratorial items (the latter being trivially zero for the group of participants who had not received any of these cards).

**Figure 2:**
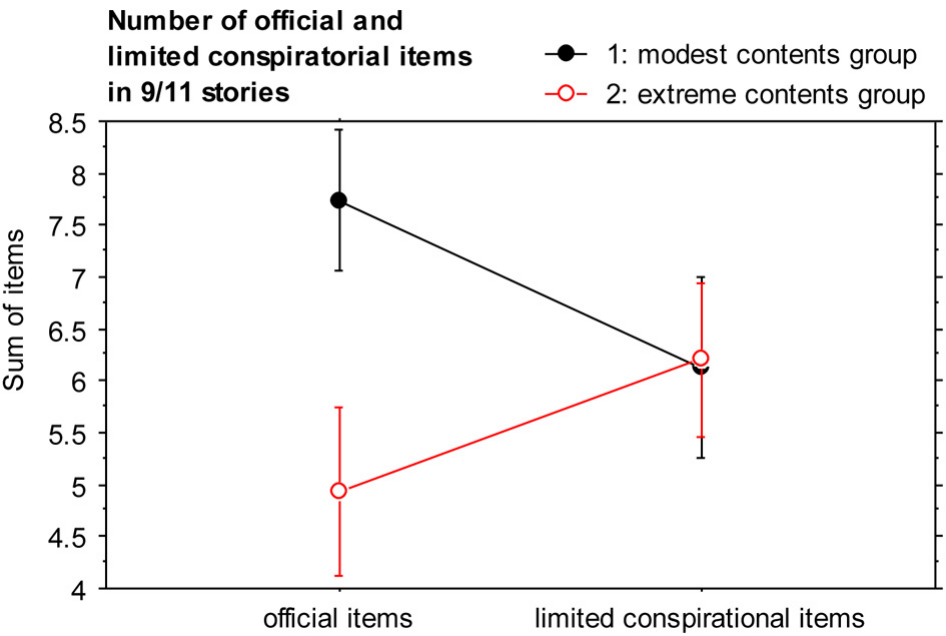
**Comparison of number of official and limited conspiratorial items for both experimental groups in the to be generated 9/11 stories.** Error bars indicate ±1 SEM (standard error of the mean).

In the modest conspiratorial condition, participants on average selected 7.7 out of 13 official items (*SD* = 2.6) and 6.8 out of 13 limited conspiratorial items (*SD* = 3.3) to construct a 9/11 story (Figure 2). On average, 12.8 items were used, with a range from 6 to 23 items. When the full set was available, there were 4.9 out of 13 official items selected on average (*SD* = 3.2), 6.2 limited conspiratorial items (*SD* = 2.4) and 3.9 unlimited conspiratorial items (*SD* = 3.7). 15.8 items were used on average, with a range from 6 to 30 items.

With a One-Way Analysis of Variance (ANOVA), we tested if number of items selected from the official as well as from the limited conspiratorial item pool (these numbers being the dependent variables) differed between the two groups. The difference in the number of official items selected was significant, *F*_(1, 28)_ = 6.92, *p* = 0.0137, η^2^_p_ = 0.198, with *M* = 7.7 (*SD* = 2.6) for the official and *M* = 4.9 (*SD* = 3.2) for the limited conspiratorial item pool, whereas we found no difference in the number of selected limited conspirational items, *F*_(1, 28)_ < 1, *p* = 0.95, *n.s.*

Importantly, the different composition of items for the single stories did not lead to different plausibility levels, thus potential acceptance of the regarding stories. When analyzing the plausibility ratings of the stories, we could not reveal any difference between the *extreme contents group* (*M* = 4.0, *SD* = 0.5) and the *modest contents group* (*M* = 3.8, *SD* = 0.9), *F*_(1, 28)_ < 1, *p* = 0.45, *n.s.*).

## Discussion

### Theoretical discussion

People had to generate their own stories for one of the most dramatic events of contemporary history. The available building blocks were limited to a number of statements taken from the real world, i.e., reflecting the official version of 9/11 as well as mild and extreme conspiratorial views. The stimuli were selected to match a set of categories that was identified to be typical for conspiracy theories.

The small number of categories and the three-level design confined the stories' content. Yet, mathematically the participants had the opportunity to build one out of over eight billion possible stories (already when the structure, i.e., the item order of the laid story, is not regarded). Furthermore, there was no time restriction.

Our results indicate that people construct a plausible explanation for an important event by integrating all pieces of information available, even if this information implies a huge conspiracy.

While one would expect a going to extremes in a discussion of several persons, the significant drop in the number of canonical items shows that a shift of the bounds of plausibility already begins in an individual's mind. Notably, there was no time pressure, and the time people used can be considered well above the duration of usual media coverage. Consequently, we would not consider this effect as a heuristics in the sense of a cognitive shortcut. Indeed, the effect appeared as a result of a thorough consideration of information. The result was not a single-best answer, but a coherent story.

We asked German people to construct a 9/11 narrative; we might expect the stories' content to be influenced by the participants' home country and, going hand in hand, the individual concern with the 9/11 aftermath. However, we wanted to induce active story construction, and for our German sample we could be sure every participant knew of this event; and at the same time we could be fairly sure there was no personal involvement—in a sense that a participant might have known one of the 9/11 victims personally.

As items were taken from real-world sources, they were not matched in terms of representativeness for a given category or factor level. Thus, there will very likely have been differences in *conspiratorialness* within the groups. Additionally, there were different levels of mutual exclusion: some extremely conspiratorial items were not compatible with their official counterpart (and vice versa); for example, a controlled detonation ruled out the planes as ultimate cause for the collapse of the towers. Other items, however, were mutually consistent; for example, a government lying about Pearl Harbor can be in accordance with an Al-Qaeda attack. Further research has to show if a matching of items is possible; and if it is desirable, as heterogeneous and in part mutually exclusive information is characteristic of real-world opinion formation.

Another promising research question would be the stability of generated narratives. For example, if participants are asked to construct a story again 1 or 2 days later: will they produce the same plot?

The shift from a moderate toward an extreme conspiracy did not come with a decline of self-perceived story plausibility. What we did not test, however, was to which extent the participants identified with their story. Would they cling to it when they were confronted with the necessity to act; for example, when they would be asked to defend their narrative against critical questions?

One could object that participants were limited by the story fragments available, particularly in the non-extreme condition, and thus not able to produce the “perfect” conspiracy they would have looked for. If so, however, we would have expected a lower plausibility rating on average for this group; or, alternatively, a drop in mildly conspiratorial items when the full set was presented, with the number of canonical items not affected.

While the method and the results presented here could undoubtedly be optimized, they indicate that extreme positions in an alleged conspiracy foster the active acquisition of that conspiracy. This indicates a danger we will discuss in the light of one of the most heated public debates in Germany of recent years.

### Germany is abolishing itself: the practical dangers of absurd statements

Sarrazin's (2010) book *Deutschland schafft sich ab* (*Germany is abolishing itself*) was a “blockbuster”—in a double sense. On the one hand, it was a huge success in terms of publicity, spearheading Germany's non-fiction bestseller list for 21 consecutive weeks (Buchreport.de, n.d.), making it the most successful book about politics from a German author of the decade (Media Control, 2010). On the other hand, it has mined public debate about the integration of people with migration background until today.

In his book Sarrazin devises a scenario which displays all of our criteria for a conspiracy theory: While Germany's population is diminishing, Muslim minorities keep growing due to constantly high birth rates (*odd event*). Thus, Sarrazin predicts that the “real” Germans—cultural pureness can be seen as the esoteric *myth*-element here—will soon be outnumbered by the offspring of immigrants from Muslim countries. Highly fertile, yet unwilling to adopt our value system, these people (*group of conspirers*) are secretly (*non-transparency*) taking over the German society, gradually reorganizing it in accordance with their religious beliefs. Sarrazin's line of argument mixes facts, opinions and anecdotes from very different areas and levels of life and knowledge (*evidence*). Most controversial were his crude assumptions of an “IQ score being 15 points higher” (Sarrazin, 2010, p.93) among Jews of European origin; as well as his claim that we “become more stupid on average for mere reasons of demography” (p.100), as Muslim immigrants, in Sarrazin's argumentation, would lower society's general intelligence level. Last but not least, Sarrazin claims that the truth about all this is being suppressed by excessive political correctness in public debate and that this self-imposed censorship is a result of collective feelings of guilt dating back to the “Third Reich” (*publicity*).

Many protagonists in the debate refuted the extreme statements about a linkage between religion, fertility and religiously determined intellectual brilliance. Yet, they admitted that Sarrazin had made some important points about migration in general (as critically discussed, for example, by Lau (2009), when Sarrazin's views had become public for the first time). Notably, the book review rated *helpful* by most other users at the British online bookstore amazon.co.uk, reads as follows: “… yes there are elements that most people will find hard to agree with no matter how persuasively argued but that shouldn't detract from the vast majority of what is being argued in the book” (Thinkforachange, 2010).

Our question here is not if these radical aspects of Sarrazin's book had been a means of promotional success, which seems beyond doubt: he got prime-time attention for months. The validity (and non-validity) of his assumptions has been discussed extensively, for example in Foroutan (2010). Also, the social dimension—has there been a taboo that Sarrazin has dared to break, or has this alleged taboo just been an excuse for some to spread xenophobic attitudes—is not in focus here.

On basis of the findings of our empirical study, we have good reason to believe that the presence of rather extreme statements shifts peoples' cognitive bounds when they construct their opinion about complex political events: they will tend to construct a more radical view when such information is offered. In this case: even if people won't adopt the view of Jewish intelligence DNA, the presence of this statement—say, while reading the book or while listening to a debate on TV—might result in a more extreme personal narrative. Adaptation research points us in the direction of the possible reason for this: As soon as we perceive and process extreme items, we integrate them into our mental representation (e.g., Strobach and Carbon, 2013) yielding adaptations toward the new items (e.g., Carbon, 2011), thus the whole narrative gets more extreme. What has been shown by these authors to work in the visual domain, seems to hold for verbal, semantic information, too.

So a conspiracy theory (in the sense outlined here) bears many dangers: the complex and anecdotic reasoning immunizes against falsification. Extreme constituents attract attention and polarize the debate; and they also might induce a shift of people's individual explanatory constructs toward a conspiratorial plot. In sum, a flavor of oddness might not be a weakness of such theories, but indeed an integral part and enabler of their persuasive power.

### Conspiracies and reptile political leaders

Extreme and sometimes absurd statements seem to be an ingredient of many conspiracy theories. But what role do reptile aliens and flying Nazi saucers play in conspiracy theories? Are such statements merely included for dramatic effect in order to attract our attention, or do they really affect what we *believe* in the end?

We have shown that the presence of rather extreme statements does have an effect on people's story construction. The “official” view becomes of lesser importance. Moderate items are disregarded, and in turn extreme statements are integrated. With a case study of Sarrazin's book *Deutschland schafft sich ab* (*Germany is abolishing itself*) we illustrated the danger of a theory containing established facts, speculations and rather crude opinions.

We focused on the constructive nature of forming an opinion. Such an opinion was seen as a *story*—a system of coherent information—answering key questions related to a given event or process: Why did it happen? Who is responsible? Who is affected?

We deem this view crucial for research on conspiracy theories. One does not simply perceive such a theory to accept or refute it. One will rather match this theory with one's own *eventuality space*, that is, all things one deems possible. In the end, the eventuality space might be *recalibrated* to incorporate new facts just as recent findings on the adaptivity of memory representations have shown (e.g., Carbon, 2011; Carbon and Ditye, 2011). In turn, the person might come up with a new (conspiracy) theory that shares some, but not necessarily all elements of the original theory. As Leman and Cinnirella (2007) has already noted, biases and heuristics play an important role. While he focused on the cause-effect-relationship, we considered the scope of information as an influencing factor on the frame of plausibility.

It is these dynamics of reception, alteration and propagation that account for the many-faceted phenomenon we call conspiracy theory. The cognitive effort, i.e., considering information in the eventuality space, might be rewarding and satisfying in itself; just like an aesthetic experience or a mental exercise (cf. Muth and Carbon, 2013). Unlike a crossword puzzle, however, reception and propagation of a conspiracy theory allow for intercommunion. Yet, as many participants reported afterwards, constructing a story can ultimately be *fun*.

These results might also explain why some conspiracy theories are believed—one might think of reptile aliens governing important nations in disguise of familiar political leaders—, although they seem stark mad to outsiders. Given the mechanism found here holds for an ongoing, long-term cycle of information seeking and opinion formation, it might be possible that a small but constant shift toward an extreme will not arouse the truth-seeker's suspicion.

As a next step, we will take a closer look at the process of story construction, e.g., by letting participants *think aloud*. Right now, we do not know what motifs guide the individual's constructive process. With a larger sample, we will also compare the structure of the generated narratives to identify whether there are certain aspects, respectively content categories that are more likely to be influenced by the presence of extreme opinions. Taking a closer look at individual differences (Are there predictors for people who will fall for this effect? Are there people who might even be deterred by extremist testimonies, thus responding with a shift in the opposite direction?) is on the agenda, too.

### Conflict of interest statement

The authors declare that the research was conducted in the absence of any commercial or financial relationships that could be construed as a potential conflict of interest.

## Footnotes

^1^*PRISM* is an US government codename for an extensive data collection effort, allegedly organized within an intelligence operation based on electronic surveillance procedures. The existence of PRISM was leaked by IT developer Edward Snowden in June, 2013.
